# HTLV-1 p30^II^: selective repressor of gene expression

**DOI:** 10.1186/1742-4690-1-40

**Published:** 2004-11-24

**Authors:** Patrick L Green

**Affiliations:** 1 Department of Veterinary Biosciences, The Ohio State University, Columbus, OH 43210, USA; 2 Molecular Virology, Immunology, and Medical Genetics, The Ohio State University, Columbus, OH 43210, USA; 3 Center for Retrovirus Research, The Ohio State University, Columbus, OH 43210, USA; 4 Comprehensive Cancer Center, Arthur G. James Cancer Hospital and Solove Research Institute, The Ohio State University, Columbus, OH 43210, USA

## Abstract

Human T-lymphotropic virus type-1 (HTLV-1) is a complex retrovirus that causes adult T-cell leukemia/lymphoma (ATL) and is implicated in a variety of lymphocyte-mediated disorders. HTLV-1 pX ORF II encodes two proteins, p13^II ^and p30^II ^whose roles are beginning to be defined in the virus life cycle. Previous studies indicate the importance of these viral proteins in the ability of the virus to maintain viral loads and persist in an animal model of HTLV-1 infection. Intriguing new studies indicate that p30^II ^is a multifunctional regulator that differentially modulates CREB and Tax-responsive element-mediated transcription through its interaction with CREB-binding protein (CBP)/p300 and specifically binds and represses *tax/rex *mRNA nuclear export. A new study characterized the role of p30^II ^in regulation of cellular gene expression using comprehensive human gene arrays. Interestingly, p30^II ^is an overall repressor of cellular gene expression, while selectively favoring the expression of regulatory gene pathways important to T lymphocytes. These new findings suggest that HTLV-1, which is associated with lymphoproliferative diseases, uses p30^II ^to selectively repress cellular and viral gene expression to favor the survival of cellular targets ultimately resulting in leukemogenesis.

## 

The complex sequence of events set in motion by human T-lymphotropic virus type 1 (HTLV-1) to cause proliferation and ultimately transformation of T lymphocytes is beginning to be unraveled. Only recently has it become clear that viral encoded proteins, the so-called "accessory" gene products of this complex retrovirus, play an integral role in the pathogenic process. In addition to the structural and enzymatic gene products, HTLV-1 encodes regulatory and accessory proteins from four open reading frames (ORF) in the pX region between *env *and the 3' long terminal repeat (LTR) of the provirus [1,2]. The well studied Rex and Tax positive regulators are encoded in the ORF III and IV, respectively. Rex plays a critical role in nuclear export of unspliced or singly spliced viral mRNA [3,4]. Tax orchestrates multiple interactions with cellular transcription factors and activates transcription from the viral promoter and modulates the transcription or activity of numerous cellular genes involved in cell growth and differentiation, cell cycle control, and DNA repair [5,6]. Recent studies have indicated novel roles for pX ORF I and II gene products in the replication of HTLV-1 [7-9]. Although the study of these gene products were largely by-passed by virologists until the mid 1990's, they intensified when infectious molecular clones provided the tools to better understand their role *in vivo*. Both HTLV-1 pX ORF I and II mRNAs have been detected in infected cell lines and blood leukocytes from HTLV-1-infected subjects including ATL and HAM/TSP patients [10,11]. Also, immune responses of HTLV-1 infected patients and asymptomatic carriers indicate that these proteins are expressed in vivo [12-14].

Molecular clones of HTLV-1 with selective mutations of ORF I and II have revealed the requirement of p12^I ^and p13^II^/p30^II ^in the establishment of infection and maintenance of viral loads in a rabbit model of infection [15-17]. The nuclear and nucleolar localizing p30^II ^has minimal homology to transcription factors Oct-1 and -2, Pit-1, and POU-M1 [18-21]. In addition, the protein co-localizes with p300 in the nucleus and physically interacts with CREB binding protein (CBP)/p300 and differentially modulates cAMP responsive element (CRE) and Tax response element-mediated transcription [21,22]. Intriguing recent reports also indicate a post-transcriptional role of HTLV-1 p30^II ^and HTLV-2 p28^II^(homologous protein encoded in the HTLV-2 pX ORF II region), in repressing the export of *tax/rex *mRNA from the nucleus [23,24]. Thus, it appears that HTLV-1 has yet another multifunctional protein with transcriptional and post-transcriptional roles in regulating viral gene expression.

Microarrays are important tools to gain insight into changes in gene expression profiles of virus-infected cells. This approach has been primarily used to investigate gene expression in HTLV-1-immortalized/transformed cell lines or in cells from ATL patients [25-29]. In the report by Michael et al. [30] the authors used the Affymetrix U133A human gene chip to test the role of HTLV-1 p30^II ^as a regulator of gene expression in Jurkat T cells. They identified alterations in gene expression profiles unique to cell cycle regulation, apoptosis, and T lymphocyte signaling/activation. Although p30^II ^expression, as might be expected from earlier reports, resulted in a general repressive pattern of gene expression, their data indicated that the viral protein selectively spared or enhanced NFAT, NFκB, and AP-1 mediated transcription in T cells undergoing co-stimulation. Signaling pathways primarily affected by p30^II ^as measured by luciferase reporters included both NFAT and NFκB, which increased from approximately 3 to 11 fold, depending on co-stimulatory treatment. Overall, this study supports earlier reports on the repressive role of HTLV-1 p30^II ^in gene expression [21-24] and reveals new potential mechanisms by which p30^II ^may play a role in HTLV-1 replication (figure 1). The effects of p30^II ^appear to overlap or counteract the influence of other HTLV-1 regulatory proteins like Tax or other accessory proteins such as p12^I^. Further studies to test if these proteins act coordinately or synergistically will undoubtedly shed light on this issue. It is possible that HTLV-1 employs selective use of these viral proteins during various stages of the infection to promote cell proliferation, a hallmark of the diseases associated with the deltaretrovirus family. Whatever the outcome of these studies, it is clear that "accessory" proteins, like p30^II^, may have "essential" roles in the life cycle of HTLV-1.

## Abbreviations

HTLV-1, human T cell lymphotropic virus type-1

ATL, adult T cell leukemia

HAM/TSP, HTLV associated myelopathy/tropical spastic paraparesis

ORF II, open reading frame II

LTR, long terminal repeat

CRE, cAMP responsive element

CREB, cAMP response element binding protein

NFAT, nuclear factor of activated T cells

NFκB, nuclear factor kappa B

AP-1, activator protein 1

## Competing Interests

The author(s) declare that they have no competing interests.

**Figure 1:**
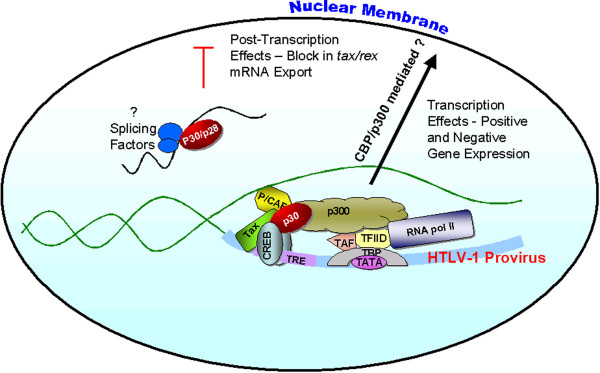
Model for HTLV-1 p30^II ^transcriptional and posttranscriptional gene regulation. The cell nucleus surrounded by the nuclear membrane and key components are shown. p30^II ^can directly interact with CBP/p300 and modulate transcription of viral and/or cellular genes. At low concentration p30^II ^may stabilize the transcription complex and potentiate transcription, whereas a high concentration it may compete for limited amounts of CBP/p300 and repress gene expression. p30^II ^(as well as the homologous p28^II ^of HTLV-2) specifically binds *tax/rex *mRNA and block its export, reducing Tax and Rex and ultimately repressing viral gene expression. This interaction may be directly linked to splicing factors and splicing and/or the juxtaposition of specific exon/exon junction sequences. Thus, p30^II ^is a multifunctional protein with transcriptional and post-transcriptional roles in regulating viral and/or cellular gene expression.
